# Identification of small molecule inhibitors for influenza a virus using *in silico* and *in vitro* approaches

**DOI:** 10.1371/journal.pone.0173582

**Published:** 2017-03-08

**Authors:** Juliann Nzembi Makau, Ken Watanabe, Takeshi Ishikawa, Satoshi Mizuta, Tsuyoshi Hamada, Nobuyuki Kobayashi, Noriyuki Nishida

**Affiliations:** 1 Department of Molecular Microbiology and Immunology, Graduate School of Biomedical Sciences, Nagasaki University, Sakamoto, Nagasaki, Japan; 2 Leading Program, Graduate School of Biomedical Sciences, Nagasaki University, Sakamoto, Nagasaki, Japan; 3 Nagasaki Advanced Computing Center, Nagasaki University, Bunkyo-machi, Nagasaki, Japan; Cornell University, UNITED STATES

## Abstract

Influenza viruses have acquired resistance to approved neuraminidase-targeting drugs, increasing the need for new drug targets for the development of novel anti-influenza drugs. Nucleoprotein (NP) is an attractive target since it has an indispensable role in virus replication and its amino acid sequence is well conserved. In this study, we aimed to identify new inhibitors of the NP using a structure-based drug discovery algorithm, named Nagasaki University Docking Engine (NUDE), which has been established especially for the Destination for GPU Intensive Machine (DEGIMA) supercomputer. The hit compounds that showed high binding scores during *in silico* screening were subsequently evaluated for anti-influenza virus effects using a cell-based assay. A 4-hydroxyquinolinone compound, designated as NUD-1, was found to inhibit the replication of influenza virus in cultured cells. Analysis of binding between NUD-1 and NP using surface plasmon resonance assay and fragment molecular orbital calculations confirmed that NUD-1 binds to NP and could interfere with NP-NP interactions essential for virus replication. Time-of-addition experiments showed that the compound inhibited the mid-stage of infection, corresponding to assembly of the NP and other viral proteins. Moreover, NUD-1 was also effective against various types of influenza A viruses including a clinical isolate of A(H1N1)pdm09 influenza with a 50% inhibitory concentration range of 1.8–2.1 μM. Our data demonstrate that the combined use of NUDE system followed by the cell-based assay is useful to obtain lead compounds for the development of novel anti-influenza drugs.

## Introduction

The control of influenza virus infection is a major public health concern due to the significant morbidity and mortality it causes through seasonal epidemics and pandemics. Human influenza infections are mainly caused by influenza A virus (IAV) and influenza B virus (IBV), however, IAV causes the majority of influenza infections. Seasonal influenza vaccines are the mainstay tools for influenza prevention; but due to the high mutation rates of influenza viruses, these vaccines need to be updated annually. IAV undergoes frequent genetic reassortment and this may potentially lead to new strains emerging that are capable of causing a global pandemic, as experienced with the novel H1N1 pandemic in 2009 that resulted in more than 284,000 deaths worldwide within the first year of the pandemic [1]. Therefore, antiviral drugs are also required to help reduce the spread of an emerging influenza pandemic. M2 inhibitors (amantadine and rimantadine) and neuraminidase inhibitors (oseltamivir, zanamivir, peramivir and laninamivir) have been developed and used widely. Recently, however, the effectiveness of these drugs has been limited by the rapid emergence of drug-resistant strains [2–4]. A 2007 seasonal influenza A(H1N1) virus has been reported to have acquired oseltamivir resistance and spread globally within a 12-month period [5–10]. Since 2011, clusters of oseltamivir-resistant A(H1N1)pdm09 influenza virus have been detected in Australia, USA, Japan and China [11,12]. Some of the A(H1N1)pdm09 influenza oseltamivir-resistant variants possess additional mutations associated with increased viral fitness and transmission [13,14]. It is of great concern that a novel strain with highly virulent characteristics and resistant to existing antiviral drugs may emerge. Therefore, new drugs with novel mechanisms of action are urgently needed.

The IAV nucleoprotein (NP) is highly conserved [15,16], and has versatile functions during the virus replication cycle. It is a major component of viral ribonucleoprotein (vRNP); and NP monomers interconnect to form a double-helical oligomer that encapsidates viral RNA and binds to heterotrimeric polymerase (PA, PB1 and PB2) [17]. Crystal structure analysis has revealed that NP exists as trimers and consists of head, body and tail regions [17,18]. The tail loop consisting of amino acid residues 402–428 is crucial in trimerization. The tail loop projects away from the NP body domain and inserts into the tail-binding pocket of neighboring monomers [17,18]. Multiple interactions between the tail loop and tail-binding pocket, especially a salt bridge between R416 of the tail loop and E339 of the tail-binding pocket, contribute to the formation of higher-order NP structures [17–19]. Based on these structural studies, the tail-binding pocket has been suggested as a possible drug target [17,18]. Furthermore, in reconstitution experiments, deletion of the tail loop and mutation of R416 or E339 inhibited the formation of NP oligomers and impaired vRNP activity, resulting in inhibition of virus replication [17,20]. Thus, disruption of NP-NP interactions is considered a reasonable strategy for the development of novel anti-influenza drugs and has been explored [20]. A E339-R416 salt bridge inhibitor, a 1,3-thiazole-4-carboxamide derivative, was recently discovered by rational drug design and was found to effectively disrupt NP trimerization and suppress viral replication [20]. Additional antiviral strategies targeting NP include induction of NP aggregation by nucleozin [21,22] and inhibition of the NP-RNA interaction by naproxen [23]. Other compounds including RK424 [24], mycalamide A [25] and 3-mercapto-1,2,4-triazole derivatives [26] have also been reported to target NP. Taken together, NP appears to be a promising target for drug therapy, although the reported compounds are not yet approved for clinical use.

Structure-based drug discovery (SBDD) has proven to be efficient in the discovery of effective drugs for the treatment of various diseases [27]. SBDD employs an understanding of the molecular basis of disease, the three-dimensional structure of the biological target protein and *in silico* analysis of ligand-target interactions, offering the advantage of target-based selection of lead compounds. We recently reported successful SBDD approach for prions using a novel binding simulation program, named the Nagasaki University Docking Engine (NUDE) [28], which was developed for an original Graphics Processing Unit (GPU)-based high-speed supercomputer, the Destination for GPU Intensive Machine (DEGIMA). Using this novel *in silico* docking simulation system in the current study, we identified new anti-influenza agents targeting the IAV NP tail-binding pocket, and selected a series of 4-hydroxyquinolinone compounds that potently suppressed virus replication in a cell-based assay.

## Materials and methods

### *In silico* screening

*In silico* screening was performed using an original docking simulation program NUDE, designed to run in the GPU system [28]. Algorithm employed by NUDE is based on the evolutionary Monte Carlo techniques [29], and we use an empirical free energy model for evaluating the binding energy of a ligand. The problem domain is defined as a six-dimensional search, i.e., positions and rotations that the ligand can take relative to the target protein. In this study, the EMC algorithm was terminated after 8 generations. We used a custom chemical compound library consisting of 9,430 compounds (Sigma-Aldrich, Japan) which characterize their molecular weights and Log *P* values ranging from 110.12 to 545.64 (median: 311.44) and -7.53 to 13.78 (median: 3.25), respectively. Each compound was screened with 200 conformations at a maximum, which were generated by the Open Babel software [30], followed by energy minimization with the GAFF force field [31]. In our docking simulation, the X-ray structure of IAV NP was downloaded from the Protein Data Bank (PDB code: 2IQH) [17] as the receptor, and a cubic space (26 × 26 × 26Å) that included S165, E339, D340 and A387, was selected as the search region. These four amino acid residues have been reported to play an essential role in the IAV NP-NP interaction [17], and are essential for virus replication [32–34]. The calculation was carried out using the DEGIMA supercomputer of the Nagasaki Advanced Computing Center constructed with more than 100 GPUs.

### Cells, viruses and chemicals

Madin-Darby Canine Kidney (MDCK) cells, a kind gift from Dr. Kyosuke Nagata (Tsukuba University, Japan) were maintained in Eagle’s minimum essential medium (MEM) purchased from Wako Pure Chemical Industries, Ltd (Japan), supplemented with 5% fetal bovine serum (FBS) from Life Technologies (Australia), and 100 units/mL penicillin and 100 μg/mL streptomycin (Penicillin-Streptomycin Mixed, Nacalai Tesque Inc, Japan) at 37°C in 5% CO_2_. IAV A/Aichi/2/68 (H3N2) was kindly supplied by Dr. Jiro Yasuda (Nagasaki University). IAV A/Virginia/ATCC2/2009 (H1N1) (ATCC^®^VR-1737^™^) and IBV B/Lee/40 (ATCC^®^VR-1535^™^) were obtained from the America Type Culture Collection (Manassas, VA), and IAV A/Puerto Rico/8/34 (H1N1) and A/WSN/33 (H1N1) were prepared as described [35]. Viruses were propagated in MDCK cells at 37°C, except for A/Virginia/ATCC2/2009 (H1N1) which was grown at 35°C, in 5% CO_2_ for 3 days. Infections with A/Puerto Rico/8/34 (H1N1), A/Aichi/2/68 (H3N2) and A/Virginia/ATCC2/2009 (H1N1) were performed in the presence of 8.3 μg/mL of trypsin (Nacalai Tesque Inc). Culture supernatants were harvested and stored at -80°C. Virus titer was determined by the 50% tissue culture infective dose (TCID_50_) assay [36]. Oseltamivir phosphate (F. Hoffmann-La Roche Ltd, Switzerland) and zanamivir (LTK laboratories, Inc., St. Paul, MN) were dissolved in phosphate-buffered saline (PBS) and dimethyl sulfoxide (DMSO) (Nacalai Tesque Inc), respectively. Hit compounds and their analogs were purchased from Sigma-Aldrich, 2 mg/mL in DMSO. NUD-1,(N-(2-cyanophenyl)-1,2-dihydro-4-hydroxy-2-oxo-1-pentyl-3-quinolinecarboxamide), molecular weight of 375.4 and Log *P* of 4.75) was a gift from the Center for Bioinformatics and Molecular Medicine, Nagasaki University. The compound was dissolved in DMSO to a stock concentration of 2.5 mM. Naproxen (Sigma-Aldrich) was dissolved in PBS to a concentration of 10 mM. All chemical compounds were stored at -20°C until use.

### Cell-based screening of hit compounds using a crystal violet assay

MDCK cells (3 × 10^4^ cells/well) were seeded in 96-well tissue culture plates and incubated for 24 h at 37°C. Compounds (2 mg/mL in DMSO) were diluted 100-fold with MEM vitamin (MEM containing 1% of 100× MEM vitamin; Invitrogen, Carlsbad, CA). The cells were washed with FBS-free MEM, and 68 μL of MEM vitamin was added to the cells followed by the addition of 32 μL of 100-fold diluted hit compounds and 100 μL of virus solution (1000 TCID_50_/mL of A/WSN/33 in MEM vitamin). In the first cell-based screening, each compound was tested in a single well at a concentration of 3.2 μg/mL and oseltamivir was used as a positive control at a concentration of 30 μM. Cells were incubated at 37°C for 48 h before fixing with 70% EtOH and staining with 0.5% crystal violet, as previously described [35]. The plates were air dried at room temperature and the optical density (OD) at 560 nm was measured with the Infinite M200 plate reader (TECAN, Switzerland). The percentage inhibition ratio in wells treated with compounds was calculated in reference to the uninfected untreated control. Compounds that had more than 40% inhibitory activity were subjected to second cell-based screening for determination of the 50% inhibitory concentration (IC_50_).

### Evaluation of the 50% inhibitory concentration of compounds

The dose-dependent inhibitory activity of compounds was assessed using a crystal violet assay as described above. Briefly, MDCK cells in 96-well plates were infected with 100 TCID_50_ per well of virus in the presence of increasing concentrations of compounds, oseltamivir, or zanamivir. After 48 h incubation, cells were stained with crystal violet. The percentage inhibition ratio in treated and untreated infected wells was calculated in reference to the uninfected untreated control, and plotted against the compounds concentration. IC_50_ was calculated using a linear regression analysis tool in the Microsoft excel software.

### Preparation of recombinant nucleoprotein

*Escherichia coli* strain BL21 (DE3) pLysS (BioDynamics Laboratory Inc, Japan) was transformed with pET14b-NP plasmid containing the NP gene of A/Puerto Rico/34 (H1N1) [37]. Typically, *E*. *coli* cells were grown in 100 mL of Luria broth medium at 37°C to an OD of 0.6 at 600 nm, and then induced by the addition of 1 mM of isopropyl-β-D-thiogalactopyranoside (Nacalai Tesque Inc), and further cultured for 6 h. The cultured cells were then centrifuged and cell pellets were suspended in lysis buffer (20 mM Tris-HCl (pH 7.9), 500 mM NaCl, 5 mM imidazole) in the presence of a proteinase inhibitor cocktail (Nacalai Tesque Inc). After sonication, the protein was purified using His60 Ni Superflow Resin (Clontech Laboratories Inc, CA, USA) according to the manufacturer’s instructions. Eluted samples were dialyzed in storage buffer (50 mM Tris-HCl (pH 7.4), 200 mM NaCl) and stored at 4°C.

### Surface plasmon resonance analysis

Surface plasmon resonance (SPR) analysis was performed to determine the binding affinities of the compounds and recombinant NP using the Biacore T200 system (GE healthcare Bio-Sciences, Japan) as previously described [28]. Naproxen, previously reported to bind to NP [23], was used as a positive control. Purified NP was diluted to 20 μg/mL with phosphate buffer (pH 7.5) and immobilized on a CM5 sensor chip (GE Healthcare, BR-100, 530) using an amine coupling kit (GE Healthcare, BR-1000-50). A total of 8725 response units (RU) of NP were immobilized. Compounds were dissolved in running buffer (phosphate buffer saline, 0.05% Tween 20 and 5% DMSO) at concentrations that allowed total solubilization. Different concentrations of the compounds (NUD-1 3.9–62.5 μM) and naproxen (3.9–125 μM) were injected at a flow rate of 10 μL/min at 25°C for 60 sec. The DMSO concentration was maintained at 5% in all of the solutions. A blank sensor chip was used as a control.

### Fragment molecular orbital calculation

To analyze the molecular interactions between NUD-1 and NP, fragment molecular orbital (FMO) calculation [38], was performed. The binding conformation obtained from the docking simulation was used to prepare the atomic coordinates of the complex, whereby the terminal residues of the peptide-chains in the X-ray structure (2IQH) were capped by −COCH_3_ and −NHCH_3_ for the N- and C-terminals, respectively. An energy minimization of the complex structure with classical force fields (AMBER99SB [39] and GAFF [31]) was performed using the AMBER 10 program package [40]. Using the energy -minimized structure, FMO calculation was performed, in which amino acid residues and compounds were treated as a single fragment. Interaction energies were evaluated at the Hartree-Fock (HF) level and the second-order Møller-Plesset perturbation (MP2) level with resolution of the identity approximation [41] using a cc-pVDZ basis set [42]. In this study, the PAICS program [43] was used for FMO calculation.

### Hemagglutination assay

A hemagglutination assay, as described [44], was used to determine the virus titer in the culture supernatant of cells infected with virus in the presence of NUD-1, or oseltamivir. The culture supernatant (50 μL) was transferred to 96-well microtiter plates, two-fold serially diluted in PBS and then mixed with 50 μL of 5% chicken red blood cells (Nippon Bio-Test Laboratories, Japan). The plates were then incubated at RT for 1 h. Dilution factor of the highest dilution with visible hemagglutination was multiplied by 20 to determine hemagglutination titer per mL of culture supernatant.

### WST-1 assay

The water-soluble tetrazolium salt (WST-1) assay was used to evaluate the viability of cells infected with virus in the presence of NUD-1 or oseltamivir as described previously [45], with some modifications. Briefly, MDCK cells were infected for determination of the IC_50_ as described above. Then, 48 h after infection, the culture supernatant was replaced with 100 μL of MEM vitamin solution containing 10 μL of Cell Proliferation Reagent WST-1 (Roche, Germany), as described in the manufacturer’s instructions. The cells were incubated at 37°C for 30 min and the absorbance was measured at 450 nm, using 600 nm as the reference wavelength, on the Infinite M200 plate reader.

### Time-of-addition assay

To elucidate the inhibitory mechanism of NUD-1 against influenza virus, a time-of-addition assay was performed as described [46], with some modifications. Briefly, MDCK cells (2 × 10^5^ cells/well) were seeded in 24-well tissue culture plates and incubated at 37°C. The cells were washed with MEM vitamin and infected with the virus at a multiplicity of infection of 0.001 for 1 h at 37°C. The virus solution was removed, the cells were washed and fresh medium was added. At 12 h post-infection, the culture supernatant was harvested and the virus titer was determined by the TCID_50_ assay. Different cells and/or virus treatments with the test compound were performed as follows. a) Pre-treatment of cells: test compound was added to the cells and incubated for 1 h, cells were washed and infected with virus. b) Treatment of cells during infection: test compound was added to the cells at the time of virus infection. c) Pre-treatment of virus: virus was incubated with the test compound for 1 h on ice, and then added to the cells. d) Treatment of cells after infection: cells were infected with virus and then treated with test compound at different time points over a 12 h infection period.

### Statistical analysis

The student’s t-test was performed using GraphPad software (Quick Calcs tool) to determine the statistical difference between test samples and untreated controls. A *P* value of less than 0.05 was considered statistically significant.

## Results

### Anti-influenza activity of 4-hydroxyquinolinone compounds

**Fig 1:**
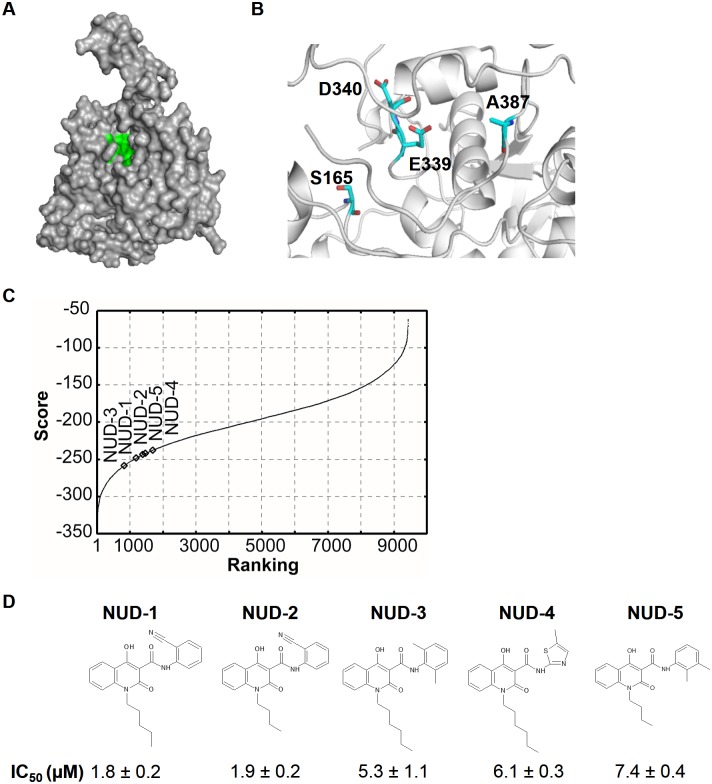
Tail-binding pocket of NP, *in silico* screening and antiviral activity of 4-hydroxyquinolinone hit compounds. (A) Crystal structure of the NP monomer (PDB code: 2IQH) represented as a surface. The tail-binding pocket of NP is highlighted in green. (B) Cartoon representation of a closer view of the tail-binding pocket of NP. Target amino acid residues S165, E339, D340 and A387 are represented as sticks. The images for A and B were generated using PyMOL software. (C) *In silico* screening was performed to identify compounds that bind to the target amino acid residues. The binding scores of 9,430 compounds were plotted as a function of the *in silico* ranking. Open diamonds indicate 4-hydroxyquinolinone compounds, NUDs 1–5, that were found to suppress virus replication in the cell-based assay (see part D). (D) Structure of NUDs 1–5 and the IC_50_ (mean ± SD) are presented. IC_50_ was obtained from three independent experiments.

**Fig 2:**
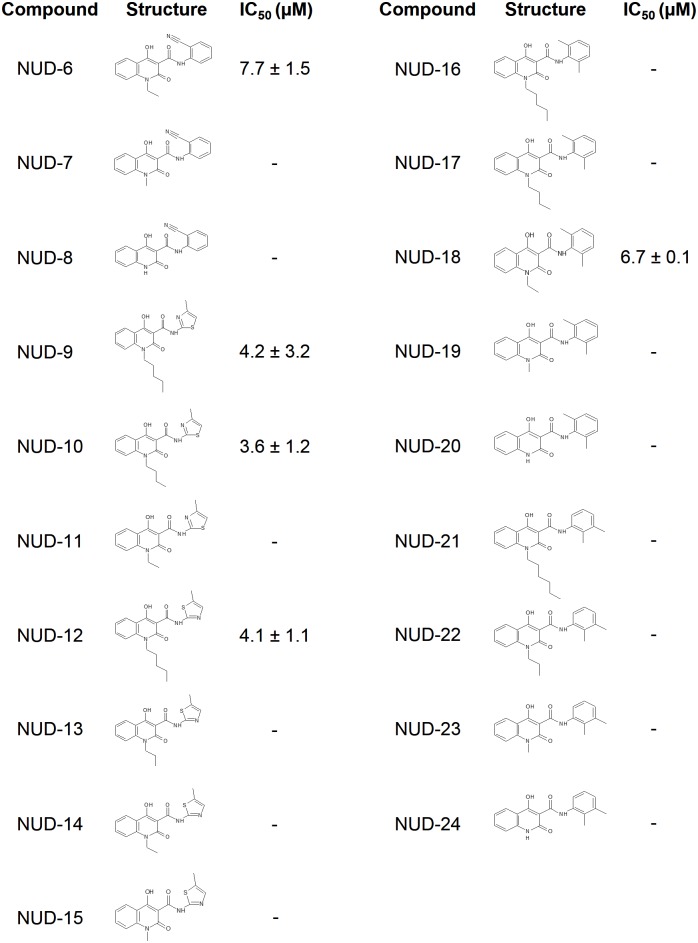
Chemical structure and anti-influenza activity of NUDs 6–24. Commercially available analogs of NUDs 1–5 were acquired and evaluated for anti-influenza activity. The results are the average ± SD from two independent experiments.

Docking simulations, using 200 conformations of each of the compounds in the chemical compound library (total number: 9,430 compounds), were performed to select compounds that bind to the tail-binding pocket of NP (Fig 1A and 1B). Hit compounds were ranked according to their binding score, which correlates with the binding affinity between the compound and the target amino acid residues (Fig 1C). We found that compounds within the top 400 were highly hydrophobic (*Log* P of more than 5); this may be due to the nature of the NP tail-binding pocket, so we expanded the first cell-based screening targets to the top 2,000 compounds to pick up some active compounds of low hydrophobicity (data not shown). Astonishingly, 115 compounds exhibited more than 40% inhibitory activity within a concentration range of 6–26 μM (3.2 μg/mL). All the 115 compounds were evaluated in a second cell-based screening, where 80 compounds showed an IC_50_ of less than 10 μM (data not shown). We found that several compounds contained the 4-hydroxyquinolinone scaffold, a core pharmacophore in several drugs [47–49]. The 4-hydroxyquinolinone compounds, termed NUDs 1–5, had similar binding scores within the range of -258 to -237 (Fig 1C), and exhibited inhibitory activity against influenza virus replication (Fig 1D). NUD-1 and NUD-2 fulfilled Lipinski’s rule of predicting drug-likeness [50], and were the most potent with IC_50_ values of 1.8 μM and 1.9 μM, respectively, while NUDs 3–5 had moderate antiviral activity and did not meet Lipinski’s rule. In an attempt to identify more potent compounds, other commercially available analogs of NUDs 1–5 were purchased and tested for anti-influenza activity (Fig 2). NUDs 6, 9, 10, 12 and 18 exhibited anti-influenza activity, albeit with less potency than that observed in NUDs 1 and 2. These data show that 4-hydroxyquinolinones are effective inhibitors of influenza virus. Since NUD-1 had the lowest IC_50_, we focused on this compound for further analyses.

### Binding analyses between NUD-1 and NP

**Fig 3:**
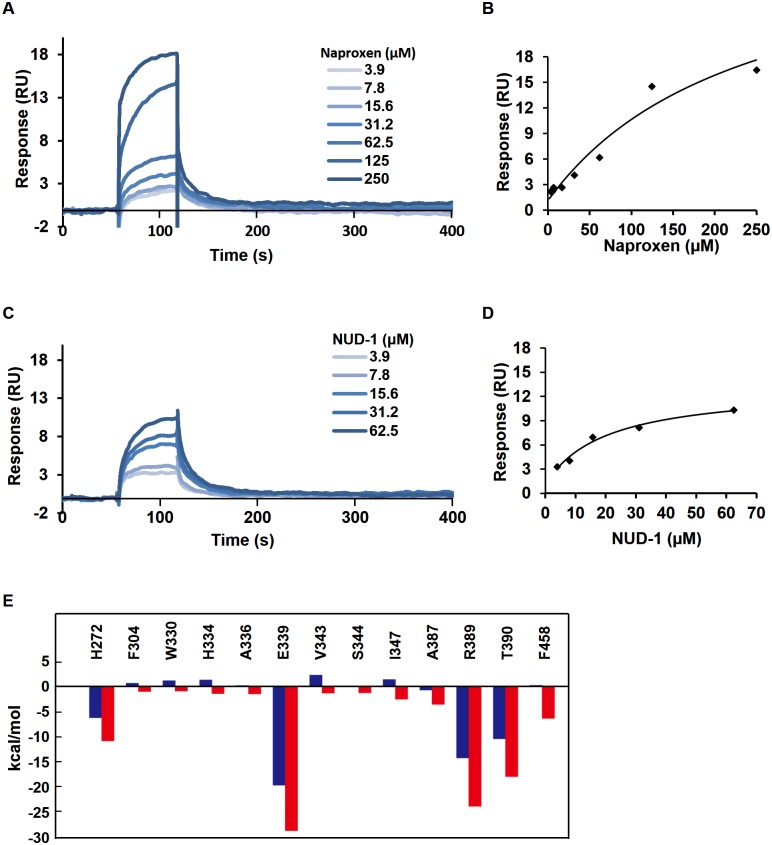
SPR and FMO analyses of NUD-1 and NP binding. Molecular interactions of NUD-1 and NP were analyzed by SPR (A–D) and FMO calculations (E). (A) Sensorgram of naproxen, (B) affinity curve of naproxen, (C) sensorgram of NUD-1, (D) affinity curve of NUD-1, (E) interaction energies of NUD-1 with NP tail-binding pocket amino acids calculated by the FMO method. The energies obtained by the HF method (blue) mainly includes electrostatic and charge-transfer interactions, and the energies obtained by the MP2 method (red) additionally includes dispersion interactions.

Molecular interactions between NUD-1 and NP were investigated by SPR analysis and FMO calculations. Prior to SPR analysis, recombinant NP was confirmed by CBB staining and western blotting (S1 Fig). Purified NP was immobilized on a sensor chip and various concentrations of NUD-1 were tested and compared with Naproxen, which has been reported to directly bind NP [23]. Naproxen showed dose-dependent binding to NP (Fig 3A), with a dissociation constant (K_D_) of 239 μM (Fig 3B). NUD-1 showed similar dose-dependent binding to NP (Fig 3C), with a K_D_ of 20 μM (Fig 3D). The interaction energies between NUD-1 and NP were calculated in Fig 3E, the interaction energies of amino acids located within 3Å of the compound are given, in which negative interaction energy represents an attractive interaction. Notably, NUD-1 displayed a highly attractive interaction with E339, a critical amino acid for NP-NP trimerization and virus replication [17,20]. Additionally, NUD-1 showed attractive interaction with H272, R389, T390 and F458, which are also reportedly involved in NP-NP trimerization [17]. Collectively, our SPR analysis and FMO calculation data indicated that NUD-1 can bind to the NP tail pocket involved in NP-NP trimerization.

### NUD-1 potently suppresses virus replication

**Fig 4:**
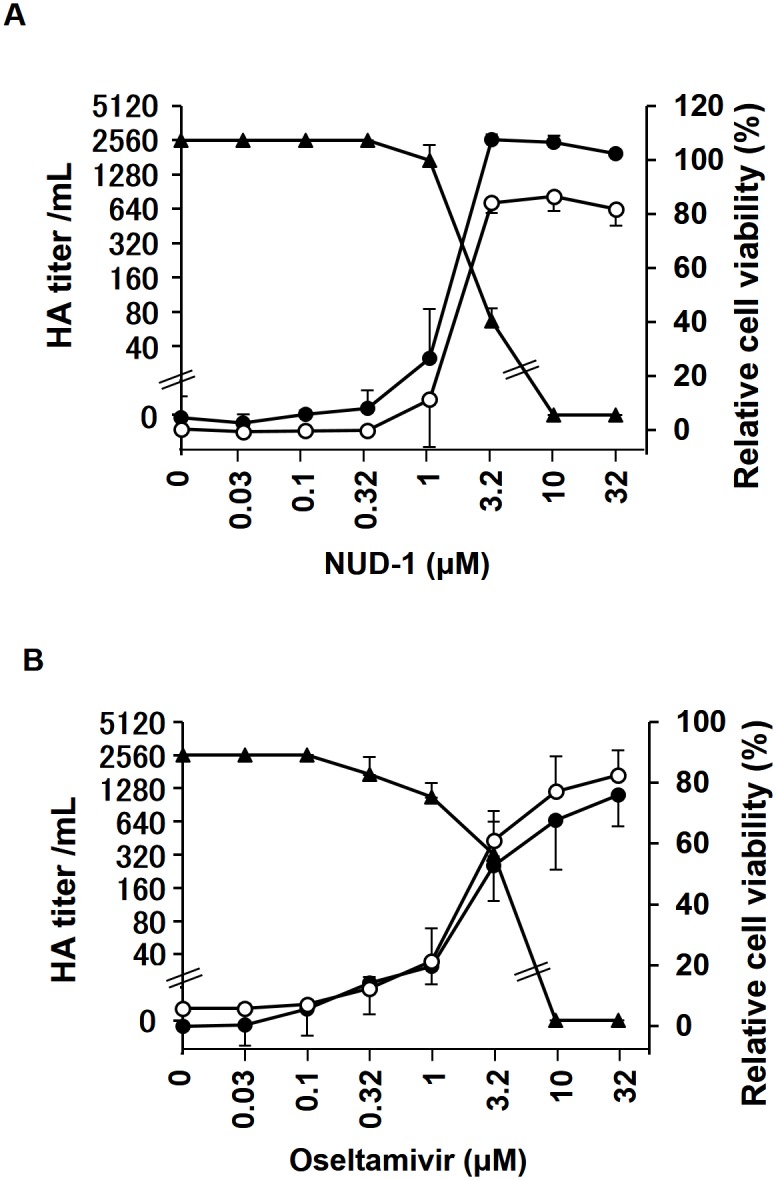
Dose-dependent inhibitory activity of NUD-1 against influenza virus. MDCK cells were infected with A/WSN/33 virus in the presence of varying concentrations of NUD-1 or oseltamivir. At 48 h post-infection, the culture supernatant was harvested for virus yield titration using hemagglutination assay. Cells viability was then evaluated by WST-1 assay and crystal violet staining. HA titer/mL (closed triangles) and relative cell viability determined by WST-1 assay (closed circles) and crystal violet assay (open circles) were plotted against the concentrations of (A) NUD-1, (B) oseltamivir.

We evaluated whether NUD-1 inhibits the viral yield in cells infected with influenza virus. MDCK cells in 96-well culture plates were infected with A/WSN/33 virus and treated with various concentrations of NUD-1 for 48 h. The culture supernatant was then harvested and the virus titer was determined by hemagglutination assay. Separately, to determine cell viability, cells were subjected to WST-1 assay followed by crystal violet staining. In Fig 4, the HA titer and relative cell viability were plotted against concentrations of NUD-1 and oseltamivir. In the absence of NUD-1, a high HA titer (2560 units/mL) and no cell viability were observed, indicating that the cells were detached from the bottom of wells by virus infection and the virus was released into the culture supernatant. In the presence of less than 3.2 μM of NUD-1, a high HA titer and low cell viability were also observed, while at concentrations of more than 3.2 μM, a low HA titer and high cell viability were observed (Fig 4A). Oseltamivir (Fig 4B) showed similar results to NUD-1 (Fig 4A). Cell viability, as determined by a WST-1 assay, was equivalent to that determined by crystal violet staining as previously reported [45]. These data demonstrated that NUD-1 and oseltamivir inhibited virus replication in a similar manner. Thus, NUD-1 potently suppressed the replication of influenza virus, and could be useful in the development of antiviral agents.

### NUD-1 inhibits the mid-stage of influenza virus infection

**Fig 5:**
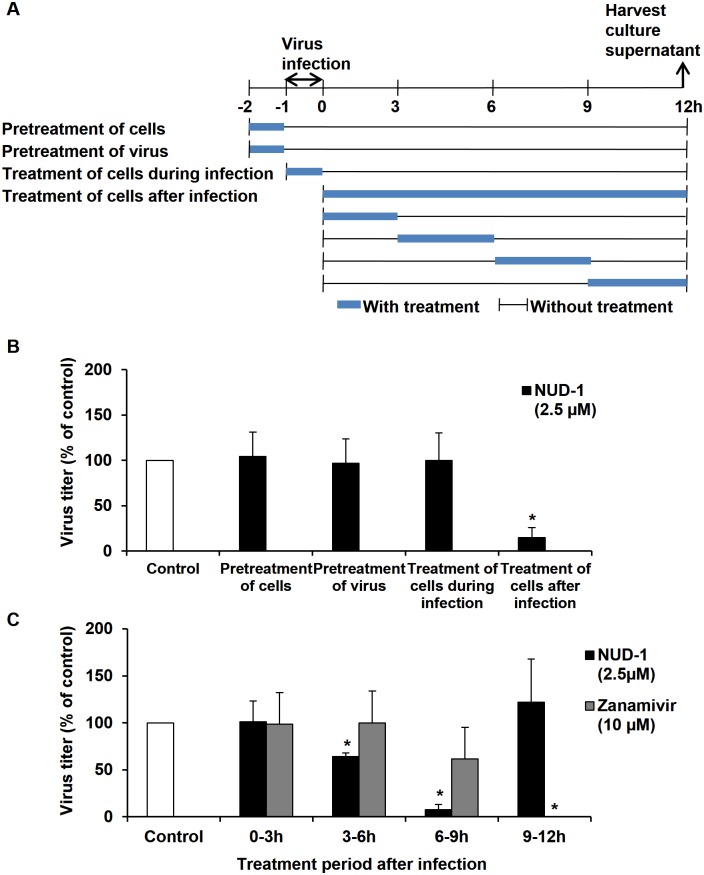
Mode of action of NUD-1 against influenza virus replication. MDCK cells were infected with A/WSN/33 virus (MOI = 0.001) and the culture supernatant was collected at 12 h post-infection for TCID_50_ assay. (A) Different cell and/or virus treatment protocols. (B) NUD-1 (2.5 μM) was added to cells before, during or after virus infection; or virus was incubated with NUD-1 before infection. C) Infected cells were exposed to NUD-1 (2.5 μM) or zanamivir (10 μM) at different time points after virus infection (0–3 h, 3–6 h, 6–9 h, 9–12 h). Virus yield for each treatment condition is represented as a percentage of the untreated control. The results are the mean ± SD obtained from at least two independent experiments. Student’s t-test was performed using Graphpad software. * indicates a *p*-value of less than 0.05.

To elucidate the inhibitory mechanism of NUD-1 against influenza virus replication, a time-of-addition assay was performed (Fig 5). MDCK cells were infected with A/WSN/33 virus using various treatment protocols (Fig 5A), and virus yield was determined by TCID_50_ assay (Fig 5B and 5C). When NUD-1 was added to the cells prior to or during viral adsorption, no reduction in viral yield was observed. Also, incubation of the virus with NUD-1 prior to infection did not reduce virus yield. However, a significant decrease in virus yield was observed when the compound was added to cells after virus adsorption (Fig 5B), suggesting that the compound inhibits virus replication events occurring after virus infection. We previously showed that treatment of cells prior to infection, during infection, and treatment of the virus prior to infection with zanamivir, inhibits virus replication [35]. Therefore, to clearly define the virus replication stage targeted by NUD-1, infected cells were exposed to the compound at different time points during the course of a single replication cycle (0–12 h post-infection, Fig 5C). Treatment of cells with NUD-1 for 0–3 h post-infection did not reduce virus yield compared with the untreated control. However, 3–6 h and 6–9 h post-infection treatment with NUD-1 resulted in a significant reduction in virus yield. The highest viral yield reduction of 92% was observed with 6–9 h treatment. NUD-1 treatment at 9–12 h post-infection did not decrease virus yield. Furthermore, no virus yield was detected when zanamivir, which inhibits the late stages of infection, was added to infected cells during the 9–12 h post-infection period (Fig 5C). These data suggested that NUD-1 inhibits mid-stage infection events of the virus.

### NUD-1 suppresses replication of influenza a viruses including H1N1 and H3N2 subtypes

Since the residues involved in NP-NP interaction in IAVs and IBVs are different [51] and the residues in the tail-binding pocket of IAV NP were used as targets for *in silico* screening, we hypothesized that NUD-1 is active against IAVs but not IBVs. NUD-1 exhibited activity against IAV strains including A/WSN/33 (H1N1), A/Puerto Rico/8/34 (H1N1), A/Virginia/ATCC2/2009 and A/Aichi/2/68 (H3N2), but not against IBV strain B/Lee/40 (Table 1). In addition, we investigated the sensitivity of these viruses to neuraminidase inhibitors, oseltamivir and zanamivir (Table 1). IC_50_ of oseltamivir against A(H1N1)pdm09 and IBV was more than 316 μM, while zanamivir showed elevated IC_50_ against IBV. Collectively, these data demonstrate the applicability of NUD-1 as an antiviral agent against IAVs including A(H1N1)pdm09 influenza.

**Table 1:** Susceptibility of influenza viruses to NUD-1 and neuraminidase inhibitors.

	IC_50_ (μM)^a^		
Influenza viruses	NUD-1	Oseltamivir	Zanamivir
A/WSN/33 (H1N1)	1.8 ± 0.2	7.4 ± 0.9	0.04 ± 0.01
A/Puerto Rico/34 (H1N1)	2.1 ± 0.1	49.5 ± 7.1	0.5 ± 0.1
A/Virginia/ATCC2/2009^b^	1.9 ± 0.2	> 316	3.2 ± 1.0
A/Aichi/2/68 (H3N2)	2.0 ± 0.9	25.6 ± 15.9	1.9 ± 0.9
B/Lee/40	> 32	> 316	7.3 ± 1.8

^a^IC_50_: 50% inhibitory concentration,

^b^Clinical isolate of A(H1N1)pdm09 influenza

## Discussion

In this study, we used an SBDD approach to identify inhibitors of IAV targeting NP, using a novel binding simulation program and *in vitro* assays. We identified a series of 4-hydroxyquinolinone compounds that effectively suppressed virus replication in infected MDCK cells (Figs 1 and 2). Analysis of binding between the most potent compound, NUD-1, and NP using an SPR assay showed that the compound binds to NP (Fig 3C and 3D). Investigation of molecular interactions using FMO showed that NUD-1 interacts with NP amino acids involved in NP-NP trimerization (Fig 3E). NUD-1 showed a strong interaction with E339, an essential amino acid in NP-NP trimerization and virus replication. Further investigations into the virus replication stage targeted by NUD-1, revealed that the compound was most effective when added to cells during the 6–9 h post-infection period (Fig 5C), a time period that correlates with vRNP assembly in the nucleus and export to the cytoplasm [52–55]. NUD-1 did not show antiviral activity when added to cells 9–12 h post-infection. Zanamivir, which targets the release of progeny virions, potently suppressed virus replication when added 9–12 h post-infection. Therefore, NUD-1 demonstrates an inhibitory mechanism different from that of zanamivir. Collectively, these results suggest that NUD-1 exerts a novel mechanism of action such as the inhibition of NP-NP interactions essential for virus replication.

**Fig 6:**
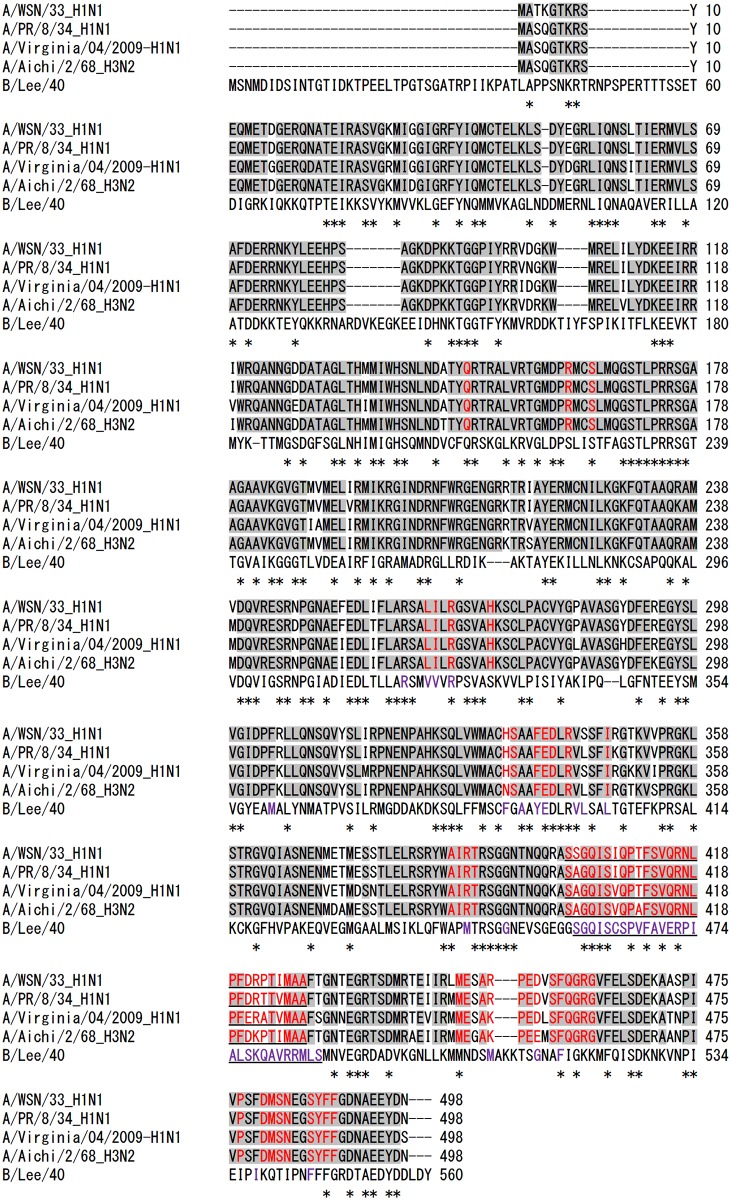
Multiple alignment of IAV and IBV NP sequences. Sequences of A/WSN/33 (AAA43452.1), A/Puerto Rico/8/34 (NP_040982.1), A/Virginia/04/2009 (ACR08603.1), A/Aichi/2/68 (AFM71861.1) and B/Lee/40 (NP_056661.1) were obtained from the NCBI protein database and aligned using Clustal Omega software. The NP sequence for strain A/Virginia/ATCC2/2009 was not available, so strain A/Virginia/04/2009 was chosen as a representative sequence for A(H1N1)pdm09 influenza. Asterisks indicate conserved amino acid residues between IAV and IBV, while conserved residues among IAV strains are highlighted in grey. Amino acid residues important for NP-NP interactions in IAV and IBV are indicated in red and purple font, respectively. Tail loop regions are underlined.

According to information from crystal structure analysis, the NPs of IAVs and IBVs are similar with head, body and tail regions. However, due to differences in NP-NP interactions, IAV NPs exist in trimers [17,18] and IBV NPs exist in tetramers [51,56]. Both proteins have a distinct tail loop that deeply inserts into the tail-binding pocket of neighboring molecules leading to self-oligomerization. Electrostatic and hydrophobic interactions between the tail loop and tail-binding pocket stabilize the formation of NP higher structures [17,51]. Amino acid residues important for these interactions in IAV [17–19] and IBV [51] are summarized in Fig 6. Conserved amino acid residues in IAV and IBV, and those conserved only in IAV are shown. Notably, NP-NP interaction amino acid residues among IAV strains are highly conserved, and differ from the NP-NP interaction residues in IBVs. In our study, NUD-1 exhibited activity against IAV strains with a narrow IC_50_ range of 1.8–2.1 μM, but activity against IBV was not determined (Table 1). Structural alignment showed that the tail loops in IAV and IBV have different orientations and could not be superimposed [51]. Thus, due to differences in IAV and IBV NP-NP interaction features, it is conceivable that NUD-1 may not bind to the NP tail-binding pocket of IBV.

The efficacy of current anti-influenza drugs is limited due to the emergence of drug-resistant viruses. We observed increased IC_50_ of oseltamivir against clinical isolate of A(H1N1)pdm09 (A/Virginia/ATCC2/2009) and B/Lee/40 influenza viruses. Although zanamivir demonstrated good antiviral activity, the susceptibility of A/Virginia/ATCC2/2009 and B/Lee/40 influenza viruses to this drug was lower than that of other viruses. These results could be explained by other studies that reported reduced efficacy of neuraminidase inhibitors against IBV [57,58], and resistance of A(H1N1)pdm09 influenza to oseltamivir [59,60]. Additionally, it has been suggested that oseltamivir exerts pharmacologic effects on the nervous system [61]. It is, therefore, important to find new inhibitors with novel mechanisms of action. Since NP-NP interactions are essential for vRNP formation, and vRNP is indispensable in virus replication, NP is a good target for drug development.

The quinoline scaffold is a privileged pharmacophore that has found application in various medicinal agents [62]. Due to the wide biological activity of quinoline derivatives, they are considered good lead compounds in the search for new drugs. One of the most extensively investigated quinoline core compounds is the 4-hydroxyquinolinone scaffold. It has found application in the development of roquinimex, as an immuno-stimulant and anti-cancer agent [47,63], laquinimod for multiple sclerosis [49], paquinimod for systemic sclerosis [48], tasquinimod for prostate cancer [64], and anti-parasitic decoquinate for coccidiosis [65]. In our study, we found that 4-hydroxyquinolinone compounds could effectively suppress influenza virus replication. This indicates that the 4-hydroxyquinolinone scaffold, through structural modifications and structure-activity relationship studies, can be improved as a potent antiviral agent.

The conventional approach to drug discovery involving high throughput screening of chemical compound libraries for biological activity is tedious, expensive and time consuming. Recently, research and pharmaceutical institutions have adopted the application of SBDD for fast and efficient discovery and optimization of lead compounds. This has been facilitated by advances in computational techniques and structural biology, enabling *in silico* analysis of the molecular interactions between ligands and their targets. SBDD has led to the discovery of various drugs including neuraminidase [66] and polymerase [67] inhibitors for influenza, proteinase inhibitors for AIDS [68,69] and hepatitis C virus [70], renin inhibitor for hypertension [71] and anticancer agents [72]. With the application of SBDD, many compounds are screened within a short time and this process is further accelerated by supercomputing technologies. For example, our DEGIMA supercomputer NUDE-based *in silico* screening system facilitates the screening of 9,340 compounds within 9 h, with each compound being screened at 200 conformations. The high number of compound conformations allows for accurate selection of compounds with high binding affinity to target, which are then evaluated for biological activity. Although we confirmed a good correlation between the binding score *in silico* and binding ability *in vitro* (S2 Fig), and some compounds with high a binding score *in silico* demonstrated antiviral activity, there was no correlation between the binding score *in silico* and the IC_50_. Thus, incorporation of pharmacokinetics prediction algorithms to the NUDE system will greatly improve the process of discovering potent drug candidates.

In conclusion, we have identified NUD-1, a 4-hydroxyquinolinone compound that potently suppresses influenza virus replication. Our data suggest that NUD-1 targets the NP tail-binding pocket leading to the inhibition of virus replication of IAVs including a clinical isolate of A(H1N1)pdm09 with reduced susceptibility to neuraminidase inhibitors. NUD-1 presents a good chemical scaffold for further development of anti-influenza drugs. Overall, initial *in silico* screening of the interaction of chemical compounds with molecular targets prior to biological activity analysis is a useful approach for the rapid prediction and selection of the most important chemical compounds.

## Supporting information

S1 Fig Purification of recombinant NP. Samples were analyzed by 10% SDS-PAGE, followed by CBB staining (A) and western blotting (B) using anti-NP antibody (GTX125989, GeneTex, Inc. (Irvine, CA)). Lysate of *E*.*coli* harboring pET14b-NP plasmid without (lane 1) or with (lane 2) IPTG induction and purified NP sample (lane 3) were used. The position of His-tagged NP (58 kDa) is indicated by arrowhead.
(TIF)

S2 Fig Correlation between the *in silico* binding score and SPR binding of NUDs to NP. The *in silico* binding score of NUDs 1–24 to NP was calculated using the NUDE system as described in the ‘materials and methods section’, and the *in vitro* binding properties were assessed by an SPR assay. Recombinant NP was immobilized on a sensor chip and 10 μM of the compounds were sequentially injected in the running buffer. The RU value has been normalized based on the molecular weight of each compound and is an average from three independent experiments. NUDs 1–24 were categorized into groups based on their structures. NUD compounds bearing cyanophenyl (NUDs 1,2 and 6–8), dimethyl phenyl (NUDs 3,5 and 16–24), and thiazole (NUDs 4 and 9–15) moieties are indicated by triangles, squares and circles, respectively. Among them, compounds having antiviral activity in cell-based assay are represented by black symbols. For all of NUD compounds 1–24, correlation coefficient was calculated.
(TIF)

S1 Table Chemical names of NUD compounds. (PDF)
