# Supplementary figures and images for: Identification of small molecule inhibitors for influenza a virus using in silico and in vitro approaches
Source: PLoS One. 2017 Mar 8;12(3):e0173582. doi: 10.1371/journal.pone.0173582 (PMC5342234; doi:10.1371/journal.pone.0173582)

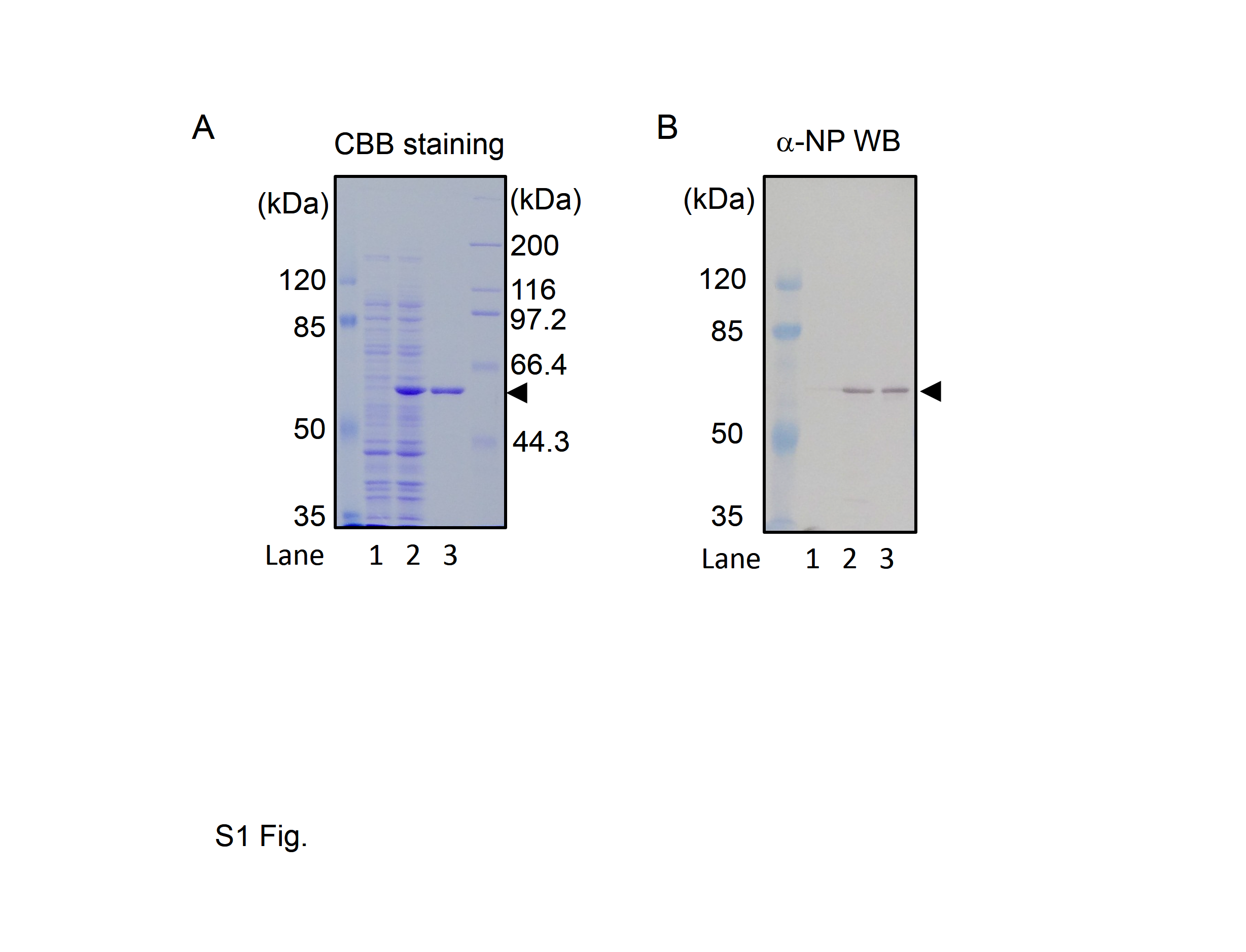

Supplement: S1 Fig — Samples were analyzed by 10% SDS-PAGE, followed by CBB staining (A) and western blotting (B) using anti-NP antibody (GTX125989, GeneTex, Inc. (Irvine, CA)). Lysate of E.coli harboring pET14b-NP plasmid without (lane 1) or with (lane 2) IPTG induction and purified NP sample (lane 3) were used. The position of His-tagged NP (58 kDa) is indicated by arrowhead. (TIF) [file pone.0173582.s001.tif]

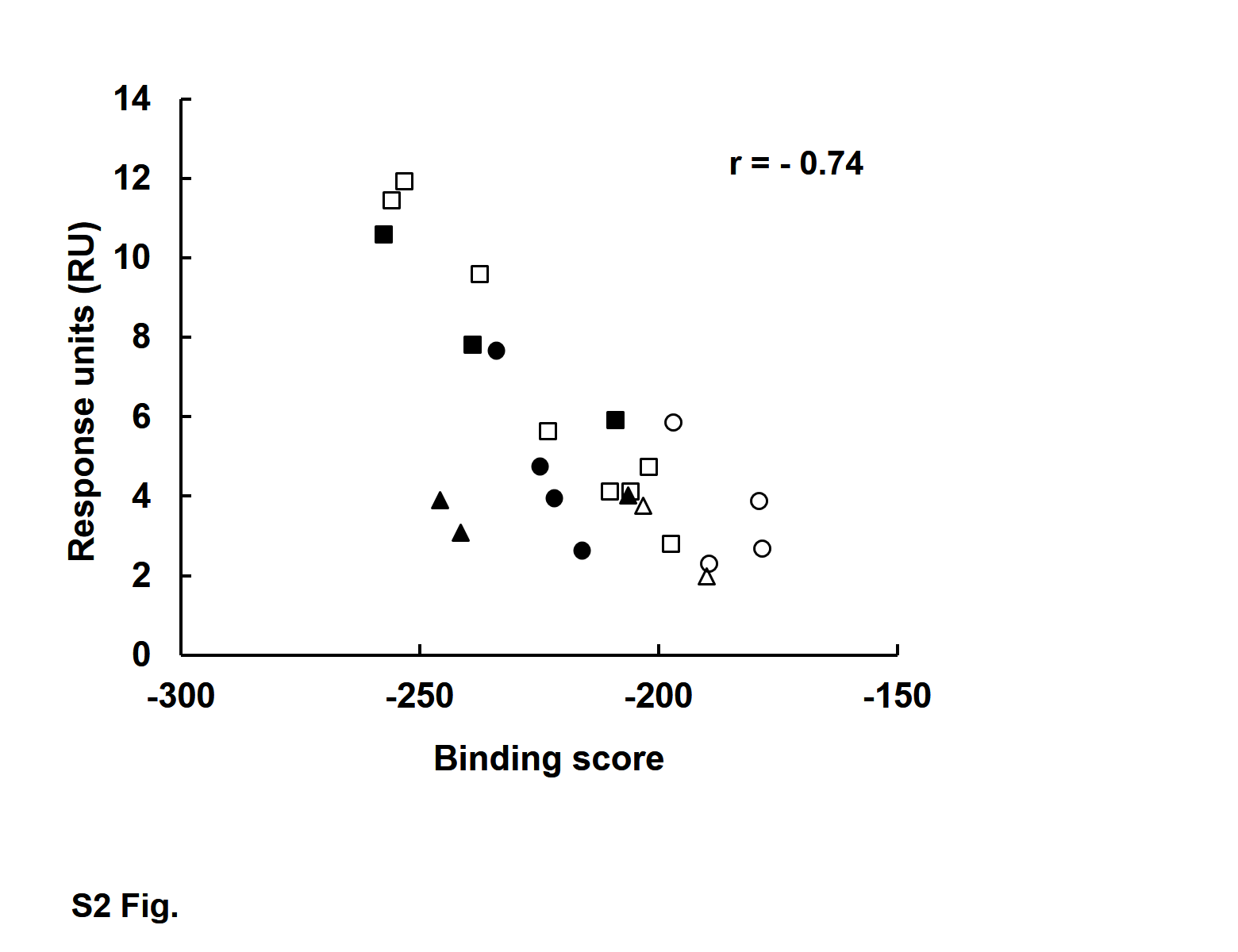

Supplement: S2 Fig — The in silico binding score of NUDs 1–24 to NP was calculated using the NUDE system as described in the ‘materials and methods section’, and the in vitro binding properties were assessed by an SPR assay. Recombinant NP was immobilized on a sensor chip and 10 μM of the compounds were sequentially injected in the running buffer. The RU value has been normalized based on the molecular weight of each compound and is an average from three independent experiments. NUDs 1–24 were categorized into groups based on their structures. NUD compounds bearing cyanophenyl (NUDs 1,2 and 6–8), dimethyl phenyl (NUDs 3,5 and 16–24), and thiazole (NUDs 4 and 9–15) moieties are indicated by triangles, squares and circles, respectively. Among them, compounds having antiviral activity in cell-based assay are represented by black symbols. For all of NUD compounds 1–24, correlation coefficient was calculated. (TIF) [file pone.0173582.s002.tif]
