# Supplementary material for: Identification of small molecule inhibitors for influenza a virus using in silico and in vitro approaches
Source: PLoS One. 2017 Mar 8;12(3):e0173582. doi: 10.1371/journal.pone.0173582 (PMC5342234; doi:10.1371/journal.pone.0173582)
Supplement: S1 Table — (PDF) [file pone.0173582.s003.pdf]

S1 Table   Chemical names of NUD compounds

NUD	IUPAC name	SMILES
1	*N*-(2-cyanophenyl)-1,2-dihydro-4-hydroxy-2-oxo-1-pentyl-3-quinolinecarboxamide	OC=1c2c(N(CCCCC)C(=O)C=1C(=O)Nc1ccccc1C#N)cccc2
2	1-butyl-*N*-(2-cyanophenyl)-1,2-dihydro-4-hydroxy-2-oxo-3-quinolinecarboxamide	OC=1c2c(N(CCCC)C(=O)C=1C(=O)Nc1ccccc1C#N)cccc2
3	1,2-dihydro- *N*-(2,6-dimethylphenyl)-1-hexyl-4-hydroxy-2-oxo-3-quinolinecarboxamide	OC=1c2c(N(CCCCCC)C(=O)C=1C(=O)Nc1c(cccc1C)C)cccc2
4	1,2-dihydro-1-hexyl-4-hydroxy-*N*-[5-methyl(1,3-thiazol-2-yl)]-2-oxo-3-quinolinecarboxamide	s1cc(nc1NC(=O)C=1C(=O)N(c2c(cccc2)C=1O)CCCCCC)C
5	1-butyl-1,2-dihydro-*N*-(2,3-dimethylphenyl)-4-hydroxy-2-oxo-3-quinolinecarboxamide	OC=1c2c(N(CCCC)C(=O)C=1C(=O)Nc1cccc(C)c1C)cccc2
6	*N*-(2-cyanophenyl)-1-ethyl-1,2-dihydro-4-hydroxy-2-oxo-3-quinolinecarboxamide	OC=1c2c(N(CC)C(=O)C=1C(=O)Nc1ccccc1C#N)cccc2
7	*N*-(2-cyanophenyl)-1,2-dihydro-4-hydroxy-1-methyl-2-oxo-3-quinolinecarboxamide	OC=1c2c(N(C)C(=O)C=1C(=O)Nc1ccccc1C#N)cccc2
8	*N*-(2-cyanophenyl)-1,2-dihydro-4-hydroxy-2-oxo-3-quinolinecarboxamide	OC=1c2c(NC(=O)C=1C(=O)Nc1ccccc1C#N)cccc2
9	1,2-dihydro-4-hydroxy-*N*-[4-methyl(1,3-thiazol-2-yl)]-2-oxo-1-pentyl-3-quinolinecarboxamide	s1cc(nc1NC(=O)C=1C(=O)N(c2c(cccc2)C=1O)CCCCC)C
10	1-butyl-1,2-dihydro-4-hydroxy-*N*-[4-methyl(1,3-thiazol-2-yl)]-2-oxo-3-quinolinecarboxamide	s1c(cnc1NC(=O)C=1C(=O)N(c2c(cccc2)C=1O)CCCC)C
11	1,2-dihydro-1-ethyl-4-hydroxy-*N*-[4-methyl(1,3-thiazol-2-yl)]-2-oxo-3-quinolinecarboxamide	s1cc(nc1NC(=O)C=1C(=O)N(c2c(cccc2)C=1O)CC)C
12	1,2-dihydro-4-hydroxy-*N*-[5-methyl(1,3-thiazol-2-yl)]-2-oxo-1-pentyl-3-quinolinecarboxamide	s1c(cnc1NC(=O)C=1C(=O)N(c2c(cccc2)C=1O)CCCCC)C
13	1,2-dihydro-4-hydroxy-*N*-[5-methyl(1,3-thiazol-2-yl)]-2-oxo-1-propyl-3-quinolinecarboxamide	s1c(cnc1NC(=O)C=1C(=O)N(c2c(cccc2)C=1O)CCC)C
14	1-ethyl-1,2-dihydro-4-hydroxy-*N*-[5-methyl(1,3-thiazol-2-yl)]-2-oxo-3-quinolinecarboxamide	s1c(cnc1NC(=O)C=1C(=O)N(c2c(cccc2)C=1O)CC)C
15	1,2-dihydro-4-hydroxy-1-methyl-*N*-[5-methyl(1,3-thiazol-2-yl)]-2-oxo-3-quinolinecarboxamide	s1c(cnc1NC(=O)C=1C(=O)N(c2c(cccc2)C=1O)C)C
16	1,2-dihydro- *N*-(2,6-dimethylphenyl)-4-hydroxy-2-oxo-1-pentyl-3-quinolinecarboxamide	OC=1c2c(N(CCCCC)C(=O)C=1C(=O)Nc1c(cccc1C)C)cccc2
17	1-butyl-1,2-dihydro-*N*-(2,6-dimethylphenyl)-4-hydroxy-2-oxo-3-quinolinecarboxamide	OC=1c2c(N(CCCC)C(=O)C=1C(=O)Nc1c(cccc1C)C)cccc2
18	1,2-dihydro-*N*-(2,6-dimethylphenyl)- 1-ethyl-4-hydroxy-2-oxo-3-quinolinecarboxamide	OC=1c2c(N(CC)C(=O)C=1C(=O)Nc1c(cccc1C)C)cccc2
19	1,2-dihydro-*N*-(2,6-dimethylphenyl)-4-hydroxy-1-methyl-2-oxo-3-quinolinecarboxamide	OC=1c2c(N(C)C(=O)C=1C(=O)Nc1c(cccc1C)C)cccc2
20	1,2-dihydro-*N*-(2,6-dimethylphenyl)-4-hydroxy-2-oxo-3-quinolinecarboxamide	OC=1c2c(NC(=O)C=1C(=O)Nc1c(cccc1C)C)cccc2
21	1,2-dihydro- *N*-(2,3-dimethylphenyl)-1-hexyl-4-hydroxy-2-oxo-3-quinolinecarboxamide	OC=1c2c(N(CCCCCC)C(=O)C=1C(=O)Nc1cccc(C)c1C)cccc2
22	1,2-dihydro- *N*-(2,3-dimethylphenyl) -4-hydroxy-2-oxo-1-propyl-3-quinolinecarboxamide	OC=1c2c(N(CCC)C(=O)C=1C(=O)Nc1cccc(C)c1C)cccc2
23	1,2-dihydro- *N*-(2,3-dimethylphenyl) -4-hydroxy-1-methyl-2-oxo-3-quinolinecarboxamide	OC=1c2c(N(C)C(=O)C=1C(=O)Nc1cccc(C)c1C)cccc2
24	1,2-dihydro- *N*-(2,3-dimethylphenyl) -4-hydroxy-2-oxo-3-quinolinecarboxamide	OC=1c2c(NC(=O)C=1C(=O)Nc1cccc(C)c1C)cccc2
